# The alignment of law, practice and need in suicide prevention

**DOI:** 10.1192/bjb.2017.3

**Published:** 2018-04

**Authors:** Helen M. Stallman, Jeneva L. Ohan

**Affiliations:** 1 Centre for Social Change, School of Psychology, Social Work and Social Policy, University of South Australia; 2 School of Psychological Science, The University of Western Australia

## Abstract

Help-seeking is important for patients with suicidal ideation. Currently, a risk management paradigm is used with patients who express suicidality; however, this may limit support and increase stigma, reducing future help-seeking. Coping planning is proposed as a paradigm shift that overcomes these problems by focusing on patient needs and strengths.

**Declaration of interest:**

None.

There has been a plethora of research related to help-seeking and mental illness; for example, a Google Scholar search for ‘help-seeking and mental health’ provided 2.17 million results. Perhaps this is not surprising in light of the considerable efforts that have been undertaken to raise awareness of mental illness and promote help-seeking. But an important question is: why would someone with a mental illness seek professional assistance to begin with, and what is the need when he/she asks for help? Although help-seeking is an important first step in accessing all treatment, here we will focus on help-seeking from the perspective of the patient disclosing suicidality.

Suicidal ideation can be seen as a coping strategy, occurring when the person has inadequate healthy and other unhealthy strategies to cope and starts to wonder whether suicide is a solution for his/her distress.^1^^,^^2^ Disclosing suicidal ideation – rather than acting on it or ruminating about it – is a healthy coping strategy. The main message that a person who is confiding suicidality is saying is, ‘I'm having trouble coping right now. I need you to help me.’ Seen in this way, the need from help-seeking is immediate support. The disclosure comes with trust that the confidante can and will help, providing minimally-sufficient support – that supports coping, but does not undermine a person's strengths and autonomy – until the person can cope independently again.^3^

However, this message is not always what the health professional may hear, or is trained to hear. Rather, what may be interpreted instead when a person discloses suicidality is, ‘I am at risk of harming myself – now that I have disclosed this risk, you are responsible to stop that from happening.’ Managing this risk is the dominant paradigm in responding to suicidality.^4^^,^^5^ Its focus is on ensuring the patient's safety and preventing death. A risk perspective reflects a society that is motivated by a commonality of anxiety about the future, rather than a commonality of need in the present.^4^ Legally, this is reflected in mental health acts, which allow the detention of people who are judged to be at risk of harm to themselves, including suicide. The risk-focused response of the clinician, then, is to assess symptoms and manage or mitigate risk, which includes assessment of suicidal thoughts and other risk factors and safety planning (e.g.^6^^–^^8^). Within an emergency department setting, disclosure of suicidal ideation is met with a triage system that prioritises externalising behaviours and the severity of suicidality to determine the risk of harm and the urgency of treatment.^9^ Assessment of risk relates to the potential for harm without treatment, not the needs for support. Thus, the law and clinical practice are aligned.

In this common scenario, however, the needs of the patient, as expressed through seeking help and disclosure, have been neglected. Moreover, a risk management approach to assessing suicide risk actively harms the patient through stigma. Stigma is a mark of disgrace – one that reduces a person in their social status to being tainted and now shamed by society – such as through presumed dangerousness or contagion of disease.^10^ Stigma affects people with mental illness broadly, including those with suicidality; for example, people who have attempted suicide describe experiencing a range of stigmas, including being seen as contagious, attention-seeking ‘drama queens’, manipulative, incompetent, weak, dangerous and hopeless.^11^ By responding to a disclosure of suicidality with an assessment of an individual's dangerousness to himself/herself and considering coercive and isolative treatment, professionals who use the risk management approach are making efforts to keep the individual safe. However, in so doing they risk begetting or promoting these stigmas, as setting these individuals apart from others implies social unacceptability. Consistent with this concern, people who have attempted suicide in the past describe being stigmatised by health professionals. By reacting to disclosures using a risk management approach, health professionals were seen to lack empathy for and dismiss their distress, and instead overreact to the potential for danger.^11^ Not surprisingly, these individuals describe feeling mistrusted and full of shame after disclosing suicide, and are often motivated to hide any future suicidality from professionals.^11^^,^^12^ Thus, patients who disclose and feel stigmatised through being dismissed, labelled as hopeless or considered dangerous may be likely to conceal future suicidality.

Clinicians in all professions often report feeling ill-prepared to work with people who are experiencing suicidality (e.g.^13^). The cause of this anxiety and fear may be the futility of the task – risk and protective factors that clinicians have long based their assessments on have been shown to be poor predictors of death by suicide.^14^ Additionally, risk is only associated with the probability of negative outcomes and with assigning blame,^15^ which creates additional fear in many clinicians. Through the concept of ‘otherness,’ that is, conceptualising another person as substantially different from the self,^16^ the risk approach also discourages clinicians who may be feeling distressed or suicidal from seeking help, as doing so might mean that they are seen as no longer capable of being the professional. Thus, the risk management approach has the potential to harm the clinician as well as the patient.

It is time to make a change to how we approach suicidality. An alternative paradigm that has been proposed is coping planning.^1^ This is a paradigm shift from a future-focused, risk approach to a present-focused, needs approach. Coping is a universal human behaviour. Feeling suicidal ideation is a normal human coping strategy to overwhelming distress. Rather than trying to determine the incalculable risk of a single endpoint (suicide), coping planning focuses the clinician's attention on the needs of the patient (i.e., helping them cope with their distress). This involves: (1) caring; (2) collaborating; and (3) connecting.^3^ The first step involves just listening and attending to the person's distress. The second step is to collaboratively identify the person's existing coping strategies and help them strengthen their coping plan by including additional strategies or professional support. The final step is connecting the person with higher intensity support, if needed. The needs assessment could show that the patient has: (1) low needs, being able to cope independently after talking about their problems; (2) moderate needs for additional professional support that may include having *ad hoc* supports in their coping plan, such as telephone helplines or their general practitioner; or (3) high needs, requiring immediate more intensive supports.^1^ The triage system for suicidality cannot be based on the same criteria as physical illnesses.^17^ The response received at disclosure can affect the progression of the distress. Attending to distress at this point needs to be a priority to meet the needs of patients presenting for problems with coping to prevent subsequent harm.

Responding to disclosures of suicidality with coping planning has real potential to overcome some of the stigmas towards people who experience suicidality. Coping is a universal experience, including for those who experience suicidality.^1^ Attending to distress, rather than moving to the practitioner task of risk assessment, can be a minimally sufficient intervention for many people.^3^ Unlike assessments of risk for danger and the potential need for coercive treatment, viewing disclosure of suicidality as an invitation to support a patient sends a socially inclusive message that the person is capable and cared about, and that they should not feel ashamed. With the focus on caring, listening and supporting, and away from assessment of future danger, there is the potential for professionals to feel competent, rather than ill-equipped and fearful. Moreover, collaborating with the patient using the coping planning approach eliminates the ‘otherness’ that is endemic to risk management, which may also improve the help-seeking of the helpers when needed.^18^

In summary, although current practices align with the risk assessment and management work of mental health acts, neither meet the needs of patients. Practice needs to focus on meeting the needs of patients when they disclose distress to promote help-seeking and prevent stigma and suicide. Consistent with our current health practices that aim to be patient-centred, coping planning provides a strengths-focused framework to respond to patients in distress who are seeking professional support. Mental health legislation needs updating to reflect current knowledge about risk and to place patient needs at their core.

## About the author

**Dr Helen Stallman** is a Clinical Psychologist and Senior Lecturer at the University of South Australia. **Dr Jeneva Ohan** is a Registered Psychologist and Senior Lecturer at The University of Western Australia.
